# Supplementary material for: Assessing the Potential of HPV16 E6 Seroprevalence as a Biomarker for Anal Dysplasia and Cancer Screening—A Systematic Review and Meta-Analysis
Source: Int J Mol Sci. 2024 Mar 19;25(6):3437. doi: 10.3390/ijms25063437 (PMC10970635; doi:10.3390/ijms25063437)
Supplement: Supplementary file 1 [file ijms-25-03437-s001.zip › ijms-2889063-supplementary.pdf]

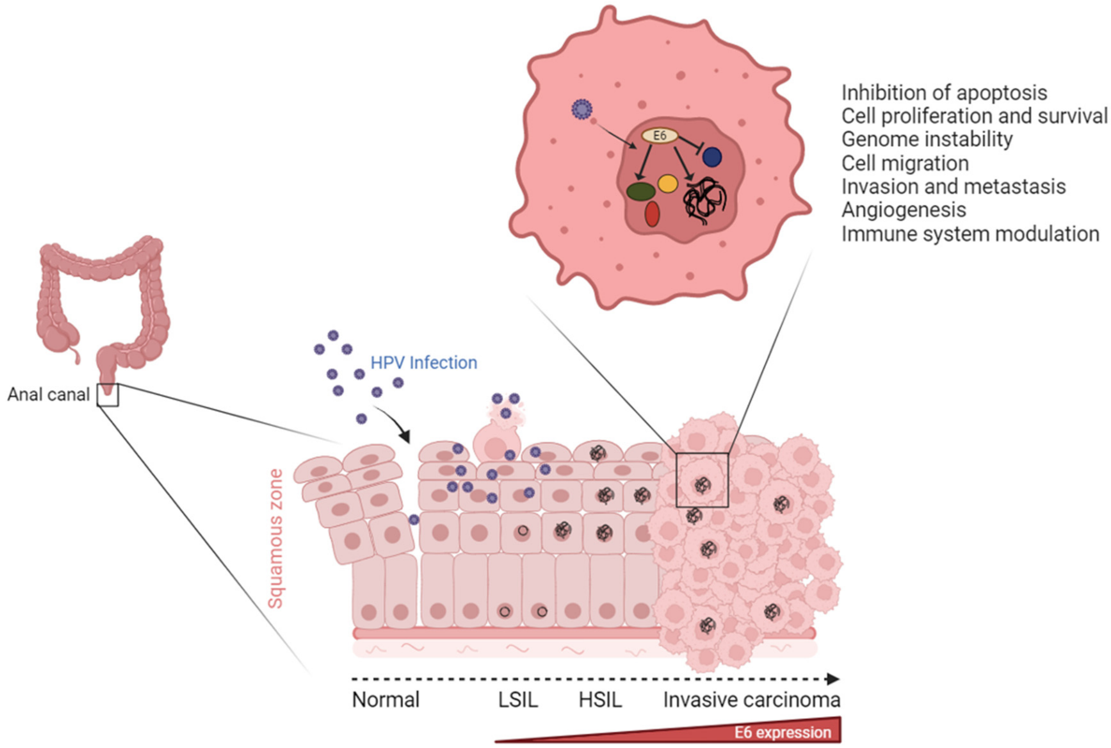

Supplementary Figure S1. Role of HPV16 E6 in the progression of HPV infection and associated disease. Human papillomavirus (HPV) initiates infection by penetrating basal epithelial cells through micro-abrasions of the anal epithelium. HPV infection may give rise to latent and subsequently productive infections. The expression of viral oncoproteins E6 is being limited due to transcriptional repression by the early protein E2. Infected basal cells undergo differentiation and migrate toward the epithelial surface, leading to the expression of late capsid genes. In low-grade intraepithelial lesions (LSIL), the viral genome replicates as an episome and becomes encapsulated in the nucleus of upper-layer epithelial cells. Shed viral particles can infect new epithelial zones or be transmitted sexually. The increased expression of E6 and E7 is associated with progression to HSIL and invasive carcinoma. Upregulation of HPV16 E6 promotes carcinogenesis by repressing tumor suppressor proteins and inducing cancer signaling pathways leading to inhibition of apoptosis, dysregulated cell proliferation and survival, genomic instability, cell migration, invasion and metastasis, angiogenesis, and genome instability. Created with BioRender.com.

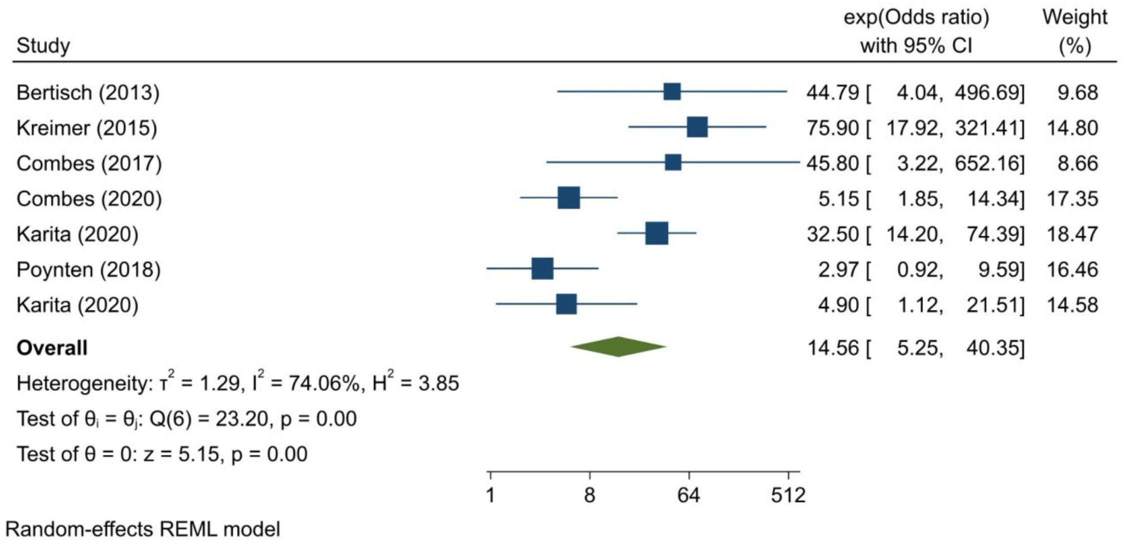

Supplementary Figure S2. Forest plot illustrating the results of a random effects model analysis of the studies included in the meta-analysis that evaluate HPV16 E6 seroprevalence in individuals with HSIL+ compared to cancer-free individuals. Odds ratios with the summary measure are displayed as the centerline of the diamond, and the associated 95% CI of the studies are shown. The contribution of each study's data to the pooled estimate is indicated by the weight (last column and size of the box). The overall degree of heterogeneity in each meta-analysis is indicated by the I^2. References: Bertisch (2013) [27], Kreimer (2015) [30], Combes (2017) [26], Combes (2020) [32], Poynten (2018) [33], Karita (2020) [31].

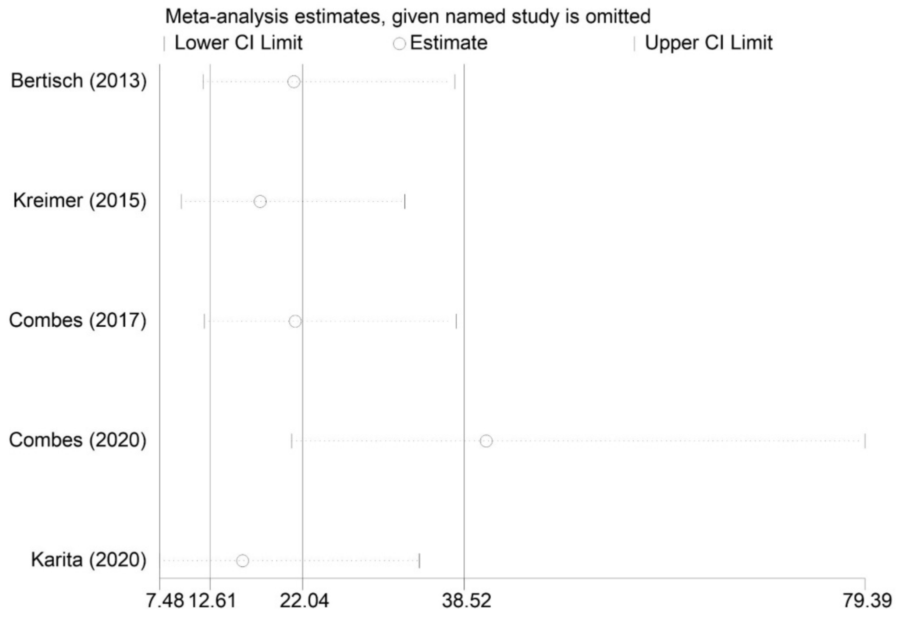

Supplementary Figure S3. Influence of each study on the estimation of the pooled effect of HPV16 E6 seroprevalence in AC. The graph visually provides the results of the meta-analysis for all studies except the study named in the specific row. The comprehensive meta-analysis result, which includes all studies, is presented at the bottom of the table and is indicated by solid vertical lines. Summary statistics are depicted as horizontal 95% CI. The full, "combined" results are shown as solid vertical lines. References: Bertisch (2013) [27], Kreimer (2015) [30], Combes (2017) [26], Combes (2020) [32], Karita (2020) [31].

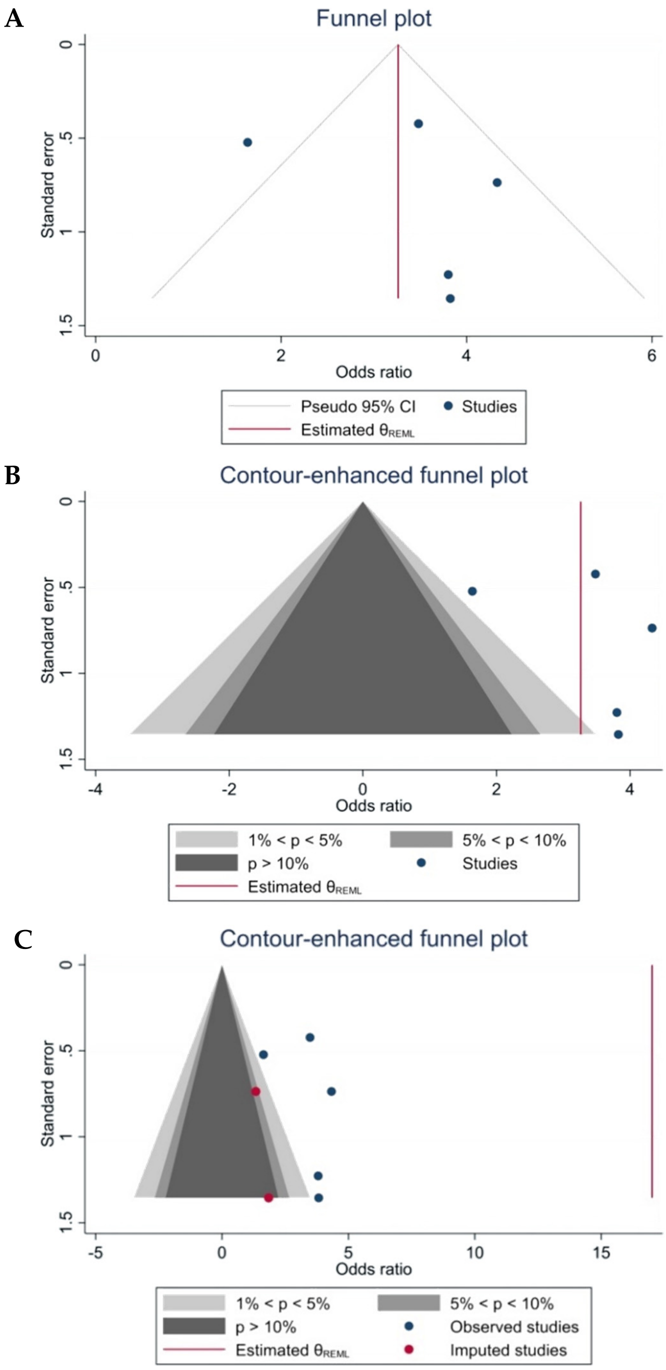

Supplementary Figure S4. Funnel plots of studies assessing HPV16 E6 seroprevalence in AC, designed to explore the presence of small-study effects often associated with publication bias. Each dot corresponds to a study-specific effect size on the x-axis, plotted against the standard errors on the y-axis. In the absence of small-study effects, the plot should exhibit symmetrical distribution (A). Additionally, contour-enhanced funnel plots are presented to explore potential asymmetry in the plot that may be attributed to publication bias, with significant contour lines at 1%, 5%, and 10% levels (B). A non-parametric "trim-and-fill" estimation method is employed to assess the number of studies potentially missing from the AC meta-analysis due to publication bias (C).

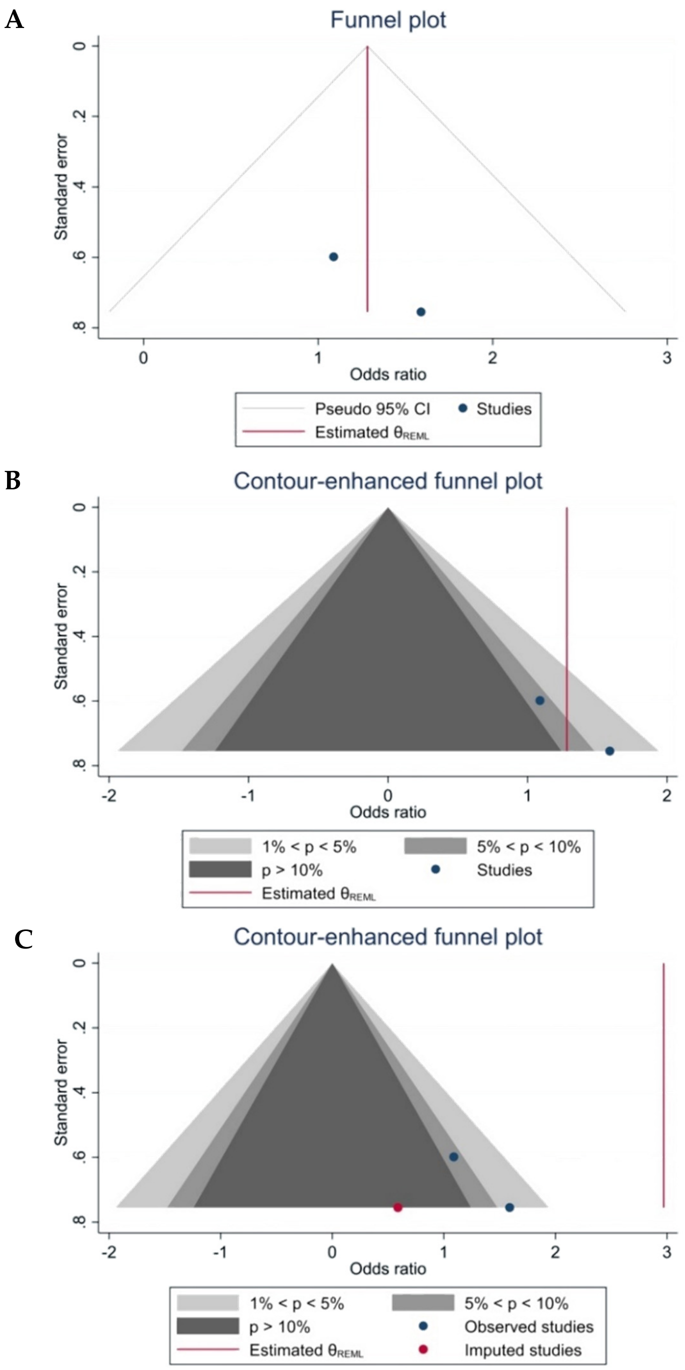

Supplementary Figure S5. Funnel plots of studies assessing HPV16 E6 seroprevalence in HSIL, designed to explore the presence of small-study effects often associated with publication bias. Each dot corresponds to a study-specific effect size on the x-axis, plotted against the standard errors on the y-axis. In the absence of small-study effects, the plot should exhibit symmetrical distribution (A). Additionally, contour-enhanced funnel plots are presented to explore potential asymmetry in the plot that may be attributed to publication bias, with significant contour lines at 1%, 5%, and 10% levels (B). A non-parametric "trim-and-fill" estimation method is employed to assess the number of studies potentially missing from the AC meta-analysis due to publication bias (C).

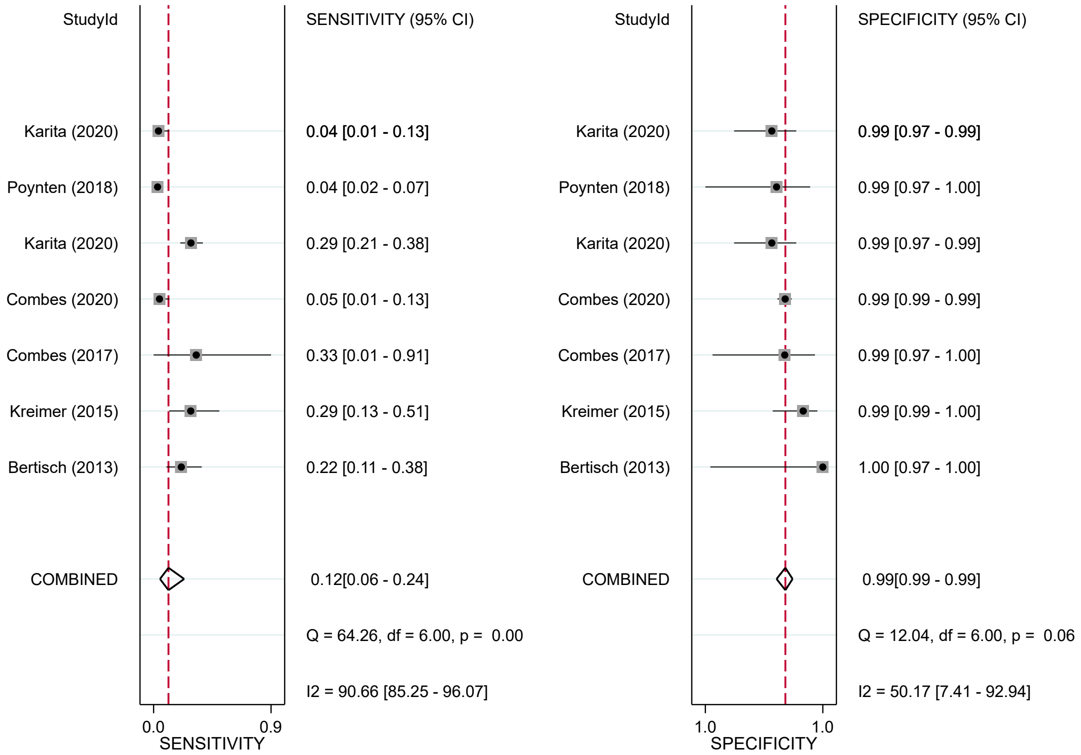

Supplementary Figure S6. Forest plot depicting pooled sensitivity and specificity for HPV16 E6 serology in relation to the HSIL+ endpoint. Each individual study is represented by a square denoting the point estimate, accompanied by horizontal lines that indicate the corresponding 95% CI. The combined sensitivity and specificity values are depicted as diamonds, with the red line signifying the consolidated point estimate. References: Karita (2020) [31], Poynten (2018) [33], Combes (2020) [32], Combes (2017) [26], Kreimer (2015) [30], Bertisch (2013) [27].

Supplementary Table S1. Sensitivity analysis.

Endpoint	Estimates	Excluding **Bertisch (2013)** [27] **Crude analysis**	Excluding **Bertisch (2013)** [27] **Adjusted analysis**
HSIL+	OR (95% CI)	11.79 (3.80-36.56)	12.95 (4.29-39.04)
AC	OR (95% CI)	23.54 (6.87-80.68)	24.43 (6.87-86.85)
	Q (df) p-value	11.76 (3) 0.00	11.68 (3) 0.009
	tau2	1.10	1.14
	I2 (%)	76.01	74.10
	H2	4.17	3.86
**Endpoint**	**Estimates**	**Including Combes (2017)** [26]	**Excluding Combes (2017)** [26]
AC	Sensitivity (95% CI)	0.20 (0.10-0.34)	0.19 (0.10-0.34)
	Q (df) p-value	17.32 (4) 0.00	17.11 (3) 0.00
	Specificity (95% CI)	0.99 (0.99-0.99)	0.99 (0.99-0.99)
	Q (df) p-value	10.34 (4) 0.04	12.44 (2) 0.01
	AUC (95% CI)	0.99 (0.98-1.00)	0.98 (0.98-1.00)
